# New insight into the adsorption behaviour of effluent organic matter on organic–inorganic ultrafiltration membranes: a combined QCM-D and AFM study

**DOI:** 10.1098/rsos.180586

**Published:** 2018-08-15

**Authors:** Xudong Wang, Danxi Huang, Botao Cheng, Lei Wang

**Affiliations:** 1 Key Laboratory of Membrane Separation of Shaanxi Province, Xi'an University of Architecture and Technology, Xi'an 710055, People's Republic of China; 2 Key Laboratory of Northwest Water Resource, Environment and Ecology, MOE, Xi'an University of Architecture and Technology, Xi'an 710055, People's Republic of China; 3 Key Laboratory of Environmental Engineering, Shaanxi Province, Xi'an University of Architecture and Technology, Xi'an 710055, People's Republic of China; 4 Research Institute of Membrane Separation Technology of Shaanxi Province, Xi'an 710055, People's Republic of China

**Keywords:** poly(vinylidene fluoride), quartz crystal microbalance with dissipation monitoring, atomic force microscope, antifouling, effluent organic matter

## Abstract

Adsorption of organic matter on membranes plays a major role in determining the fouling behaviour of membranes. This study investigated effluent organic matter (EfOM) adsorption behaviour onto poly(vinylidene fluoride) (PVDF) membrane blended with SiO_2_ nanoparticles using quartz crystal microbalance with dissipation monitoring (QCM-D) and atomic force microscopy (AFM). The QCM-D results suggested that low adsorption of EfOM and an EfOM layer with a non-rigid and open structure was formed on SiO_2_-terminated membrane surfaces. Conformational assessment showed that EfOM undergoes adsorption via two steps: (i) in the initial stage, a rapid adsorption of EfOM accumulated onto the membrane; (ii) the change in dissipation was still occurring when the adsorption frequency reached balance, and the layer tended towards a more rearranged or organized secondary structure upon adsorption onto the more hydrophilic surface. For the AFM force test, when a self-made EfOM-coated probe approached the membrane, a ‘jump-in’ was observed for the hydrophobic membrane after repulsion at a small distance, while only repulsive forces were observed for PVDF/SiO_2_ membranes. This study demonstrated that the PVDF/SiO_2_ membrane changed the entire filtration process, forming a ‘soft’ open conformation in the foulant layer.

## 1. Introduction

Ultrafiltration (UF) is an important separation technique in wastewater treatment and reclamation [1,2]. Poly(vinylidene fluoride) (PVDF) is one of the most favourable candidate substances for preparing UF membranes because of the excellent chemical resistance, thermal stability and good mechanical strength of PVDF membranes, which makes them suitable for wastewater treatment [3–5]. However, most PVDF-based membranes currently in use have low flux capacity and weak antifouling capabilities, resulting from hydrophobic properties [6,7]. It has been widely accepted that membrane fouling is a major hindrance to wide application of PVDF UF membranes for wastewater treatment [8,9].

Effluent organic matter (EfOM) has been implicated as the most important foulant in biologically treated wastewater [10,11]. Generally, commercially available alginates, bovine serum albumin (BSA) and humic materials have been used as substitutes for EfOM or natural organic matter (NOM) in membrane fouling studies. Haberkamp *et al*. [12] reported that removal of polysaccharides and proteins significantly reduced fouling and because of formation of a gel layer on the membrane surface, the accumulation of protein in the membrane pores caused much greater fouling than was expected. Susanto *et al*. [13] reported the UF fouling behaviour of polysaccharide–protein mixture solutions. However, the composition of EfOM is more complicated compared to the surrogate foulants. EfOM contains proteins, polysaccharides, amino sugars, nucleic acids, humic and fulvic acids, and cell components [14]. Some studies have compared fouling by NOM or EfOM with fouling by surrogates. Jones *et al*. [15] found that fouling of regenerated cellulose UF membranes formed by BSA was less than NOM during dead-end filtration. The similarities and differences among five organic materials were identified by Kim & Dempsey [16] according to physical and chemical characteristics, fouling during filtration and fouling mechanisms. They found that soluble microbial products were similar to EfOM in terms of fouling mechanisms and organic fractionation, but differed in molecular size distribution and specific resistance to filtration. Alginate was not representative of either EfOM or NOM. Therefore, it is important to identify the membrane fouling effect by using EfOM directly in wastewater. An evaluation of EfOM adsorption and fouling is a complex and elusive task because of the multiple interactions occurring simultaneously at the membrane surface. These might be caused by different types of interactions, such as electrostatic interactions, van der Waals forces, hydrophobic interactions and hydrogen bonding. Unfortunately, few studies have analysed the effect of membrane chemical properties on the EfOM deposited on the membrane surface during the entire filtration process. It is largely unknown how the hydrophobicity of the membrane exerts an influence on the structure and mass of the adsorbed layer on the surface. Most existing explanations appear to be simple descriptions or speculation based on experimental observations and visual comparison of the normalized permeation flux decline curves during filtration. Therefore, further investigations are required to explain the mechanism of membrane fouling on different hydrophilic membrane surfaces.

To reduce fouling, the membrane surface should provide an unfavourable environment for the deposition of foulant onto the surface and the minimum force required for foulant detachment from the surface. It is generally accepted that increasing the hydrophilic characteristics of membranes could effectively reduce membrane fouling [17,18]. As a major modification strategy, the blending of hydrophilic materials is a simple and efficient way of enhancing the hydrophilicity of PVDF membranes [19,20]. Many kinds of hydrophilic functional groups (such as ethylene glycol, –COOH, –CONH_2_ and –OH) can be used to improve the properties of membranes. Roach *et al*. [21] investigated adsorption of BSA on –OH and –CH_3_ surfaces using a combination of grazing angle Fourier transform infrared (FTIR) spectroscopy and quartz crystal microbalance (QCM). The results showed that BSA adsorption was accompanied by changes of protein conformation and was lowest on hydrophilic (–OH) surfaces. Contreras *et al*. [22] elucidated the importance of membrane surface chemical functionality in the adsorption of common organic foulants. It was found that the presence of –OH or ethylene glycol generated by surface modification would reduce membrane fouling. In general, –OH moieties can be effectively resistant to protein adherence. However, most researchers used self-assembled monolayers with different end groups on a gold surface of QCM as surrogates for actual membrane surfaces. Therefore, it is of great interest to prepare a membrane surface containing –OH groups to investigate foulant adsorption behaviour. Some hydrophilic materials containing a large number of –OH groups, such as poly(vinyl alcohol) [23–25] and poly(ethylene-*co*-vinyl alcohol) [26], have been blended with PVDF membranes. However, it should be noted that the above polymers are water-soluble, and can be washed out of the matrix during membrane preparation and operation [4]. Some researchers reported that SiO_2_ nanoparticles have many hydroxyl groups, so a small addition of SiO_2_ in the dope can allow the formation of an inorganic framework under mild conditions and incorporation of minerals into polymers, resulting in increased membrane hydrophilicity and chemical, mechanical and thermal stabilities without obviously degrading the properties of the polymers [27–30]. Thus, the PVDF membranes used in this study were modified by dispersing SiO_2_ nanoparticles uniformly in the casting solution.

For a further quantitative understanding of the effect of membrane hydrophobicity on membrane fouling, adsorption behaviour between EfOM and membranes should be determined. The QCM with dissipation (QCM-D) technique can be employed to measure minute changes of mass adsorbed on a surface *in situ* [31–33]. The additional energy dissipation-monitoring feature provides information on the structure of the adsorbed layer. The interactions between EfOM and membrane (EfOM–membrane) and between EfOM and EfOM (EfOM–EfOM) also play an important role in the adsorption phenomena. AFM has been applied in membrane fouling research to quantify intermolecular forces [32,34–36]. Bowen *et al*. [34,35] were the first to use a colloid probe to determine the adhesion forces of foulant–membrane and foulant–foulant interactions for evaluating the potential for membrane fouling. Brant & Childress [36] demonstrated that a hydrophobic polystyrene colloid had an attractive interfacial free energy at contact when the probe approached the reverse osmosis membrane, while the force for hydrophilic silica colloids was repulsive. In our previous research, a novel PVDF colloidal probe was prepared by sintering PVDF microspheres onto a cantilever, and typical organic foulant-coated colloidal probes were prepared by absorbing corresponding foulants onto the PVDF microsphere surfaces [37]. The adhesion interactions between actual organic foulants and membranes were investigated.

In this paper, we elucidated the effects of the membrane surface incorporated with different amounts of SiO_2_ nanoparticles on membrane fouling with EfOM. The interaction forces of EfOM–membrane and EfOM–EfOM were measured by AFM directly in conjunction with a novel EfOM-coated colloidal probe. QCM-D combined with membrane-coated sensor crystal was used to investigate the deposition and adsorption behaviour of EfOM on the different membrane surfaces and the structure of the EfOM adsorption layers. These results were combined with those of fouling experiments to obtain visual insight into the EfOM fouling behaviour. Understanding of EfOM adsorption over time on the different hydrophilic membrane surfaces is the first step to understanding how fouling occurs on the membrane surface. This will shed light on EfOM attachment to the surface and aggregation among EfOM on the surface, which affects the permeation rate and extent of adsorption. The insight of real-time membrane fouling by actual organic foulants will also provide critical information in predicting and preventing irreversible adsorption and fouling.

## 2. Material and methods

### 2.1. Materials

Commercial PVDF (Solef® 6020) was purchased from Solvay Co. (Germany). Poly(vinyl pyrrolidone) (PVP; K30) for pore formation was purchased from BASF (Germany). Silica (SiO_2_) nanoparticles (20–50 nm) used as an inorganic additive were purchased from Zhejiang Hong Cheng Material Co., Ltd (China). Lithium chloride (LiCl) was purchased from Tianjin Kemel Chemical Reagent Co., Ltd (China). *N*,*N*-Dimethylactamide (DMAc, greater than 99%) was obtained from Tianjin Fu Chen Reagent Company (China).

The secondary effluent of urban sewage was obtained from the fourth sewage treatment plant in Xi'an (China) and the water quality of the secondary effluent is presented in table 1.

**Table 1.** Water quality of secondary effluent of urban sewage.

	DOC (mg l^−1^)	UV_254_ (cm^−1^)	turbidity (NTU)	pH	zeta potential (mV)
secondary effluent of urban sewage	6.9	0.152	5.4	7.3	−52.3

### 2.2. Membrane preparation

PVDF/SiO_2_ blend membranes were prepared using the non-solvent-induced phase separation method. Casting solutions (table 2) were prepared using the following steps. A certain quantity of SiO_2_ nanoparticles was first added into DMAc by an ultrasonator for 2 h, which facilitated the dispersion of SiO_2_. PVDF and other additives were dissolved into the solution followed by 24 h incubation with stirring at 60°C. The resulting homogeneous solutions were then stored in a triangular beaker at 60°C to remove air bubbles. Each homogeneous casting solution was rapidly cast over a glass plate using a casting knife with a 200 µm gap, and then immersed in a bath filled with deionized (DI) water. The membranes were peeled off the glass plates and washed thoroughly with DI water to remove residual solvent prior to use in experiments. Finally, membranes were immersed in a tank containing 30 wt% glycerol aqueous solution for 24 h to prevent the collapse of porous structures.

**Table 2.** Composition of casting solution for preparation of blended membranes.

membranes	PVDF (wt%)	PVP (wt%)	SiO_2_ (wt%)	LiCl (wt%)	DMAc (wt%)
M1	17	6	0	3	74
M2	17	6	0.3	3	73.7
M3	17	6	0.5	3	73.5

### 2.3. Membrane characterization

The contact angle between pure water and the membrane surface was measured using a VCA Optima (AST Products, Inc., MA, USA). The membranes were fixed on the glass with double-sided tape and dried in a vacuum oven for 2 h before being measured. The average of at least five measurements of drops at different locations was used to obtain the contact angle for one membrane sample. A FTIR spectrometer (PerkinElmer, Japan) was employed to measure the FTIR spectra of different membranes at a resolution of 2 cm^−1^ with the spectra being obtained in the wavenumber range 4000–400 cm^−1^. The morphologies of membranes were investigated by a scanning electron microscope (SEM; S4800HSD Japan). An electrokinetic analyser for solid surface analysis (SurPASS, Anton Paar GmbH, Australia) was used to characterize the zeta potentials of the PVDF membrane surface.

### 2.4. Quartz crystal microbalance with dissipation measurements

The adherence and adsorption kinetics of the EfOM on the modified membranes were analysed in a QCM-D E1 system (Q-sence) by monitoring the change in the oscillation frequency (Δ*f*) of the membrane-coated quartz crystal sensor.

Firstly, 20-fold dilution of homogeneous casting solutions of M1–M3 membranes was prepared with DMAc. The gold-coated crystal, with a fundamental resonant frequency of around 4.95 Hz, was cleaned with 10% (w/v) sodium dodecylsulfate and then rinsed thoroughly with ultrapure water and dried using pure N_2_ gas. The cleaned gold-coated sensor crystal was fixed on the rotary platform of a spin coater (KW-4A; Institute of Microelectronics of the Chinese Academy of Sciences). The sensors were spin-coated with diluted casting solution at 1500 r.p.m. for 15 s and then the rotation speed was increased to 8000 r.p.m. for 60 s, followed by drying at 60°C for 60 min.

Before each experiment, the contact angle between pure water and the membrane-coated sensor was tested. Then, the membrane-coated sensor crystal was rinsed thoroughly with ultrapure water and dried with pure N_2_ gas. EfOM solution was used for several adsorption measurements with the membrane-coated sensor crystal. The aqueous media were injected to the sensor crystal chamber at 150 µl min^−1^ using a peristaltic pump (Ismatec, Switzerland). The temperature was maintained at 23°C. Solutions were injected sequentially to the QCM-D system in the following order: (i) DI water for 30 min; (ii) EfOM solution for 90 min; and (iii) DI water for 30 min. The variations in frequency and dissipation factor were measured for three overtones (*n* = 3, 5 and 7), but only data from overtone 5 were presented.

### 2.5. Atomic force microscopy force measurements

The colloid probes were assembled by attaching a PVDF microsphere (5 µm) onto a commercial V-shaped SiN tipless AFM cantilever end (NP-10, Bruker, Germany). Using a micromanipulator, a small quantity of double epoxy resin glue was applied to the free end of the tipless cantilever. A single PVDF microsphere was then attached to the cantilever end by the glue. Great care was taken not to coat the lower surface of the microsphere with the glue. The colloid probe was allowed to dry for several days and then stored in a glass vessel before use.

The foulant-coated probe was prepared by adsorbing the corresponding foulants onto the surface of a PVDF microsphere as reported in our previous research [37]. A similar procedure was applied to study the interaction between the EfOM-coated colloid probe and the clean modified membrane and between the EfOM-coated colloid probe and the EfOM-coated membrane surface.

The experiments were carried out at 25°C in buffer solution (1 mM NaHCO_3_, pH 7.5) in contact mode. A fouled membrane sample was rinsed at least three times with the buffer solution and installed at the bottom of the fluid cell. Determination of the force measurements commenced after the fluid cell was completely full of buffer solution. Raw data were converted from cantilever deflection and *z*-piezo position into force–distance curves. The force *F* was then normalized to the radius of the colloid probe, *R*, to obtain a comparative indicator of the membrane fouling potential [38]. For each type of membrane sample, force measurements were performed in at least 10 different locations, and more than 10 force curves were obtained at each location to minimize inherent variability in the force data. The reported adhesive force *F/R* denoted the average value of the retraction curve. The integrity of the colloidal probe was carefully examined by SEM before and after each use to ensure accuracy of the force measurements.

### 2.6. Fouling experiments and physical cleaning

Fouling experiments were performed in a dead-end filtration system using treated secondary wastewater effluent as the feed solution. The filtration protocol suggested by Ho & Zydney was adopted [39]. The testing membrane was first precompacted using 500 ml DI water at 0.15 MPa. The water flux was then measured at 0.1 MPa until the difference between consecutive records was less than 2%. The corresponding water flux was assigned as *J*_0_. The flux was calculated using the following equation:

$$J_{0}=\frac{Q}{A\times \Delta T}\;,$$

where *J*_0_ is the pure water flux (l m^–2^ h^–1^), *Q* the volume of water permeated (l), *A* the effective membrane area (m^2^), and Δ*T* the permeation time (h). Finally, 800 ml of the secondary effluent of urban sewage was filtered through the membrane at 0.1 MPa. Fouling runs were carried out at a temperature of 25°C.

Physical cleaning was conducted to determine the flux recovery of membranes, which correlated with the anti-irreversible fouling property. After filtration, the fouled membrane was rinsed with DI water for 5 min. The membrane was then cleaned with DI water at 0.1 MPa and the permeate flux was recorded. The water flux after physical cleaning was assigned as *J*_r_. The final flux recovery ratio (*J*_r_/*J*_0_) was determined from the two assigned flux values.

### 2.7. Membrane morphology observations

The top surface morphologies of the clean and fouled membranes were examined using an AFM in contact mode with an AFM probe (spring constant = 0.12 N m^–1^). The scan area was 2 × 2 µm at 512 × 512 pixels. The roughness parameter used in this paper was defined as the mean roughness, *Ra*.

## 3. Results and discussion

### 3.1. Physico-chemical properties of the composite membranes

The ability of a droplet to spread can be assessed by the contact angle of the droplet, described as the angle between the water–solid interface and the water–air interface [40]. The behaviour depends on the relative interfacial energies of the solid and the liquid surface. The water contact angles were employed to evaluate the membrane surface wettability. The contact angles of the membranes are shown in table 3. The contact angles of the membranes modified with different SiO_2_ contents were lower than the contact angle for the unmodified membrane (M1). The smaller water contact angle showed that the modified membrane was more hydrophilic. Table 3 also shows the basic characteristics of the modified membranes. Similar results were observed in previous studies [29,30]. The mechanical strength and water permeation of modified membranes were all improved.

**Table 3.** The characteristics of blend membranes and the membrane-coated sensors.

membrane	contact angle, °		average pore size, nm	zeta potential, mV	tensile strength, MPa	pure water flux, l m^−2^ h^−1^
	membranes	membrane-coated sensors				
M1	72 ± 2	68 ± 2	36	−36	2.14	689
M2	64 ± 3	61 ± 1	43	−40	2.46	773
M3	55 ± 2	53 ± 2	54	−48	2.67	987

**Figure 1.**
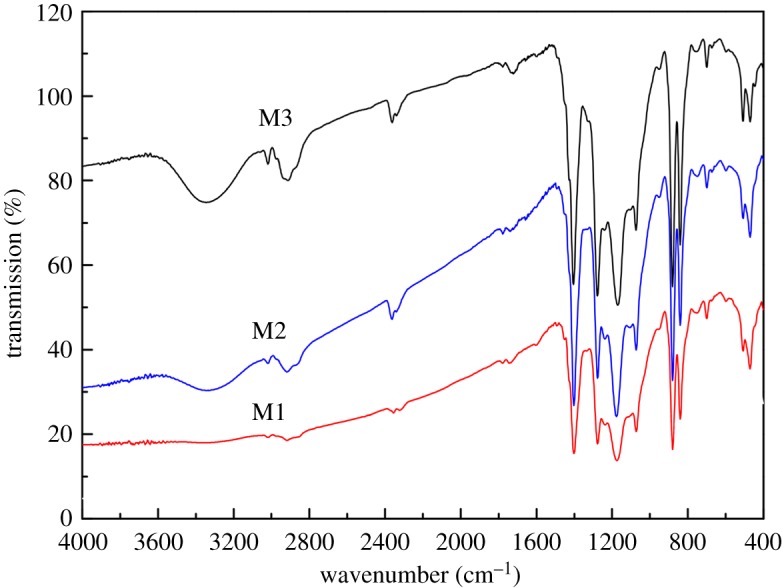
FTIR spectra of the M1–M3 blend flat sheet membranes.

Figure 1 shows the FTIR spectra of the M1–M3 blend flat sheet membranes. The strong absorption peak observed for membranes M2 and M3 at around 3450 cm^−1^ indicated a significant contribution from Si−OH. The intensities of the peaks were enhanced by the addition of SiO_2_. According to the results, it can be inferred that –OH groups can be effectively introduced into the membrane surface by the addition of SiO_2_ in this study.

### 3.2. Filtration experiments with secondary wastewater effluent

**Figure 2.**
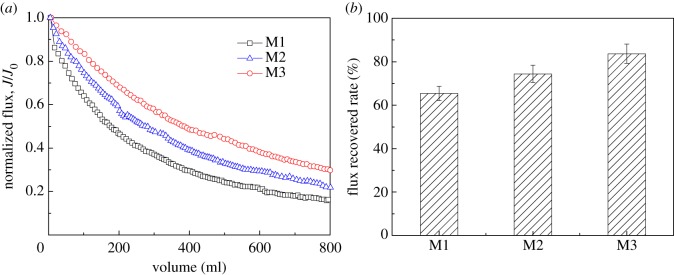
Dead-end filtration of the modified membranes with secondary wastewater effluent: (*a*) flux decline and (*b*) flux recovery rate.

Flux decline tests were performed to investigate the fouling of modified membranes by secondary wastewater effluent. The pure water fluxes of M1–M3 membranes were 689, 773 and 987 l m^–2^ h^–1^, respectively. The normalized fluxes of the blended membranes are shown in figure 2*a*, according to the flux normalization method suggested by Pieracci *et al*. [41]. The flux decline of the M1 membrane was higher than that of the membranes modified with SiO_2_ nanoparticles, indicating higher fouling of the unmodified membrane. Compared with the flux decline curve of the M3 membrane, the flux decline rate of the M2 membranes increased slightly, which may be due to the high affinity of the EfOM to the M2 membranes. The fouling reversibility of EfOM-fouled membranes is shown in figure 2*b*. The difference in the flux recoveries shown in figure 2*b* suggests that the effect of SiO_2_ nanoparticles on the fouling reversibility of the modified membranes followed the order M3 > M2 > M1. Cleaning with pure water increased the flux rate of the M3 membrane to 83.7%. Irreversible EfOM deposition was inhibited and the initial flux was readily recovered by water rinsing. The irreversible fouling of the M3 membrane may be due to the deposition of EfOM inside the membrane pores, which could not be removed by simple rinsing. Similar results were obtained by Yu *et al*. [29]. They suggested that the membranes modified with SiO_2_ particles showed improved antifouling performance. Previous research offered some simple and provisional explanations for the experimental phenomenon that membrane flux decline was slower for hydrophilic than hydrophobic surfaces. However, further information and explanations of membrane fouling mechanisms are limited, and thus further studies are required. The basis and explanations for the experiments conducted in this study are discussed below.

### 3.3. Adsorption equilibrium data analysis

**Figure 3.**
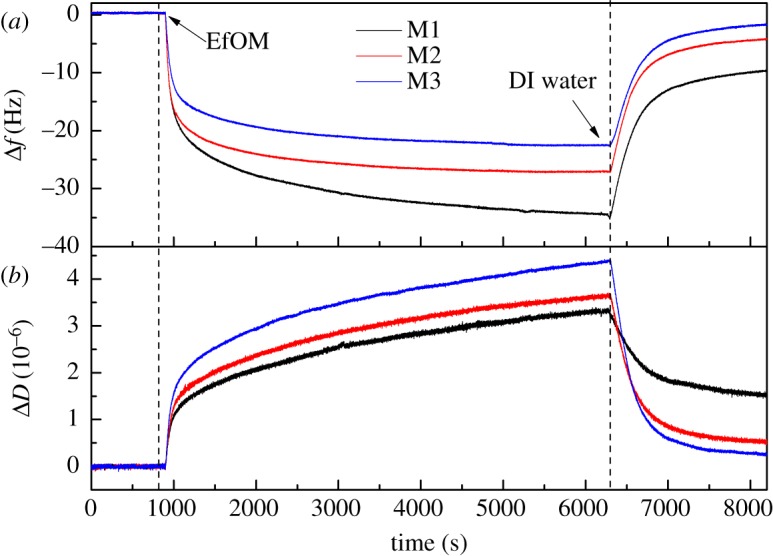
QCM-D measurement of (*a*) change in frequency (Δ*f*) and (*b*) change in dissipation (Δ*D*) versus time upon adsorption of EfOM onto the QCM-D sensor coated with M1–M3 films.

The contact angles of the membrane-coated sensors M1–M3 were similar to those of the M1–M3 membranes (table 3), indicating that they had similar surface properties. In this study, QCM-D was employed to dynamically verify the deposition of EfOM onto the modified membrane-coated sensors. The real-time shifts in frequency (Δ*f*) and dissipation (Δ*D*) at overtone 5 as a function of time for EfOM adsorption to the QCM-D sensor coated with M1–M3 are shown in figure 3. A stable baseline with DI water was obtained prior to adsorption of EfOM. At time *t* = 15 min, the secondary effluent was injected into the measurement chamber of the QCM-D. There was a decrease in frequency and increase in dissipation of membrane-coated sensors caused by immediate and rapid adsorption of EfOM. The greatest frequency shift and highest rate of frequency change were observed for adsorption onto the highly hydrophobic surface (M1). Differing foulant–surface interactions are obvious from the changes of Δ*f* and Δ*D* (figure 3). M1 showed the minimum dissipation during EfOM adsorption, which indicated a more rigid and less diffuse structure than the other treatments. The significance of this conformation change will be discussed later. Washing with DI water was performed after the EfOM adsorption experiment and frequency of all the sensors increased by about 21–25 Hz, indicating partial desorption of EfOM from the membranes. The highly hydrophilic surface (M3) desorbed significant amounts of EfOM, while the hydrophobic membrane surface showed low desorption. However, the hydrodynamic washing with DI water could not remove all the deposited foulants and the initial membrane fouling had formed.

**Figure 4.**
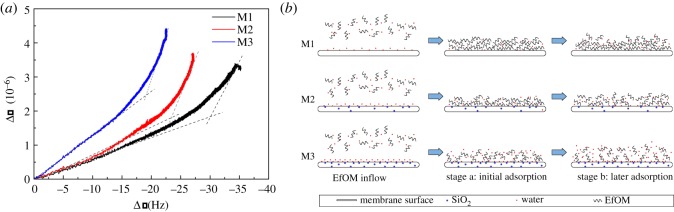
(*a*) Relationship between dissipation shift and frequency shift induced by adsorption of EfOM solution onto the QCM-D sensor coated with M1–M3 films. (*b*) A schematic diagram of the EfOM adsorption layer formed on the M1–M3 blend membrane surfaces.

The shifts in Δ*D* during EfOM adsorption to the different membrane-coated sensors are shown in figure 3*b*. Some research has reported that the slope of Δ*D* over Δ*f* gave the magnitude of the variations in the adsorbed layer fluidity, one of the main factors that damped the quartz vibration [42,43]. Thus, dissipation factors versus frequency shifts during adsorption are shown in figure 4*a*. The energy dissipation per unit change of adsorbed mass, Δ*D*/Δ*f*, was used to compare the structural characteristics of the EfOM layer on different modified membranes. A lower value of Δ*D*/Δ*f* suggested formation of a dense and compact layer, while a higher value indicated a ‘soft’ open structure and dissipative layer [44]. The Δ*D*/Δ*f* plots for the adsorption of EfOM onto M1–M3 membranes are compared in figure 4*a*. Similar to what is shown in figure 4*a*, the curves were mainly divided into two steps. (i) There was a very clear linear relationship between the change in dissipation and the shift in frequency during adsorption of the EfOM. In this stage, EfOM was rapidly adsorbed and accumulated onto the membrane surface, which was controlled mainly by the EfOM–membrane interaction. Different types of interactions, such as hydrophobic interactions and hydrogen bonding, might cause this effect. (ii) Foulants adsorbing later in the process compete for free sites that become fewer as coverage increases. The change in dissipation was still occurring when the amount of EfOM adsorbing to the membrane surface tended to balance. It was obvious that the slope of Δ*D*/Δ*f* was much steeper, indicating that the conformation of the EfOM–foulant layer was still changing over time, which caused significant energy dissipation. In the case of the M1 film, the differences in the slopes of Δ*D*/Δ*f* plots can be explained as follows: in the initial stage of rapid adsorption of EfOM onto the membrane surface, EfOM was promptly deformed to become adsorbed on the surface because of the EfOM–membrane interaction, leading to formation of a relatively rigid adsorption layer. With increasing amounts of adsorbed EfOM, there were fewer adsorption sites on the membrane surface, which meant that EfOM could not be deformed easily because of steric hindrance and the multiple interactions among EfOM components occurring simultaneously, resulting in formation of a soft adsorption layer. Compared to the hydrophobic membrane (M1), the Δ*D*/Δ*f* gradients were higher when the EfOM accumulated onto more hydrophilic membranes in both stages. This observation suggested that an EfOM layer with a non-rigid and open structure was formed on the M3 membrane surface. The data showed that EfOM had a weaker attraction to hydrophilic than hydrophobic membrane surfaces. This might be associated with the hydration of the membrane surface because of the membrane chemical modification. A schematic diagram of the EfOM adsorption layer formed on the different blend membrane surfaces is shown in figure 4*b*. Wang and co-workers [25] found that PVDF/polyethersulfone modified with 0.3% poly(vinyl alcohol) exhibited larger cohesion free energy and higher energy barrier compared to the original PVDF/polyethersulfone membrane. SiO_2_ particles are also rich in hydroxyl groups, which are very easily combined with water molecules. With the increase in SiO_2_ content, a dense hydrated layer was formed, which could effectively inhibit the initial accumulation of EfOM onto the membrane surface. The conformation of EfOM was not easily distorted upon interaction with the modified membrane surface. Water molecules penetrated into the adsorption layer gradually, accompanied by the EfOM adsorption process, so the conformation of the foulant layer became loose. Membranes modified by adding inorganic nanoparticles can influence the entire EfOM filtration process, effectively reducing the amount of EfOM adsorption but also changing the structure of the EfOM adsorption layer, improving the antifouling behaviour of the membrane.

### 3.4. Interactions between the effluent organic matter-coated probe and clean membranes

**Figure 5.**
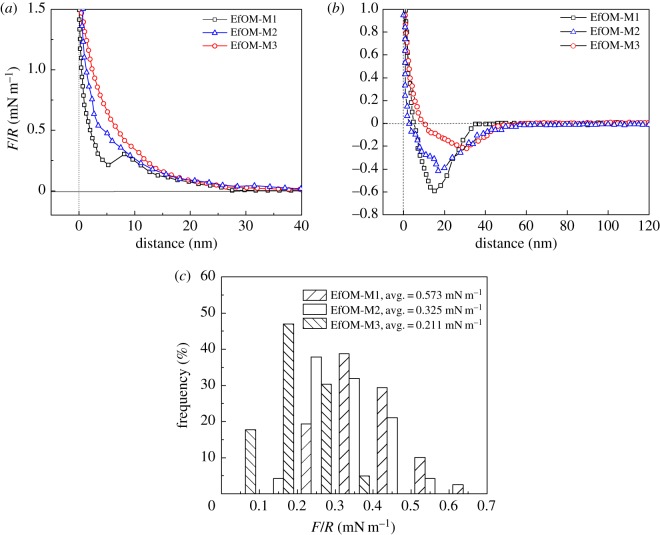
Normalized force (*F*/*R*) versus separation curves of the interaction between an EfOM-coated probe and a clean membrane: (*a*) approaching curve, (*b*) retracting curve, and (*c*) the frequency distribution of the average retraction adhesion force.

The results shown in figures 2–4 demonstrate that there was a close relationship between the fouling rate and the membrane surface properties; the more hydrophilic the membrane, the lower the fouling rate. The membrane surface was foulant-free during the initial stage of fouling; therefore, the interaction forces between the foulant and the clean membrane surface determined the initial fouling rate [45]. The force versus separation curves between the EfOM-coated probe and the clean membranes were measured. Examples of the approaching and retracting force versus separation curves for an EfOM-coated probe and the clean membranes as the SiO_2_ content varied from 0 to 0.5% are shown in figure 5. For these curves, the force was plotted against position. At positions larger than 30 nm, no force was detected. At closer distances, the membrane repelled the particles. An exponentially decaying repulsive force component was identified before the force versus separation curve increased linearly. The repulsive force was probably the electrostatic double force. The EfOM-coated probes bear a negative surface charge and they are repelled by the negatively charged membrane surface. For the M1 membrane, a ‘jump-in’ was observed after repulsion at a small distance. In this case, we speculated that the jump-in may be attributed to hydrogen bonding interactions between C–F and the EfOM-coated layer on the tip. The fact that such a jump-in was only observed for 73 out of the 120 measurements made at different locations indicated some regional variation in the membrane surface. The membrane surface variation might be a morphological variation. We believe that the jump-in was due to the interaction between EfOM and the M1 membrane surface. The jump-in disappeared after the addition of SiO_2_ in the membrane and only repulsive forces were observed for the M2 and M3 membranes. For inorganic surfaces, an extra non-Derjaguin–Landau–Verwey–Overbeek repulsion at short range was sometimes observed [46]. In addition to the electrostatic component, another force seemed to be present because of the exponential increase in the repulsive force and the disappearance of the jump-in distance. For silica surfaces, the extra short-range repulsion has been called a ‘hydration force’ [47,48]. The additional short-range force has been observed before in force–separation curves for silica in the AFM [49]. We assume that the repulsion at very small distances was probably not the only repulsive force and that a very thin water film was formed on the modified membrane surface. Other forces such as the hydration force kept the water film stable. This is likely to induce structuring of water molecules on the membrane surface through the formation of hydrogen bonds with hydroxyl groups. Other water molecules from the bulk solution would form hydrogen bonds with the adsorbed surface water. A water film was formed on the membrane surface, which could effectively prevent interaction between EfOM and the membrane surface. When 0.5% SiO_2_ nanoparticles were added in the blended membrane, the amplitude of the repulsive force was highest. These data further confirmed the results of QCM-D adsorption. When the membrane surface was hydrophobic, adsorption was even more energetically favourable because of the high affinity of EfOM to the membrane surface. Exclusion of water also allows a greater proportion of the foulant to interact with the surface, which might help to explain the greater conformational change of EfOM on the hydrophobic surface. The degree of conformational change will therefore be dependent on the EfOM–membrane surface interactions.

Contrary to the approaching curves, a normalized adhesion force was observed to pull the EfOM-coated probe off the membrane for all samples. The adhesion force generally prevented the probe from detaching from the membrane. The EFOM-modified probe experienced different levels of adhesion forces when it was retracted from the membrane surface (figure 5*b*). It appeared that, on contact, it was the ability of the EfOM-coated probe to form bonds with reciprocal groups on the membrane surface that dictated the magnitude of adhesion; however, further study is required to confirm this hypothesis. The average normalized adhesion force between the EfOM probe and the M1 membrane was 0.573 mN m^–1^, which corresponded to the minimum value in the adhesion force curve. The strong adhesion force between EfOM and M1 might be attributed to a hydrophobic attraction between the M1 membrane and hydrophobic parts of the EfOM. The contact angle decreased from 72° for M1 to 55° for M3 and the adhesion decreased slightly from 0.573 mN m^–1^ for M1 to 0.211 mN m^–1^ for M3. These results might be due to the increase in hydroxyl groups absorbed on the membrane surface with the addition of SiO_2_. For a hydrophilic surface, the bond strength between water and the membrane surface is expected to be strong. This indicated that not all water layers were removed from the surface if sufficient energy was not provided to facilitate their removal, thus preventing intimate contact between the EfOM and the membrane surface resulting in the low adhesion force. For the relatively weak hydrophilic membrane, the adsorbed water film was easily removed because of the weak bonds, allowing EfOM groups to form bonds with the membrane surface. Thus, a higher adhesion force was formed between EfOM and the membrane surface.

### 3.5. Interactions between the effluent organic matter-coated probe and fouled membranes

**Figure 6.**
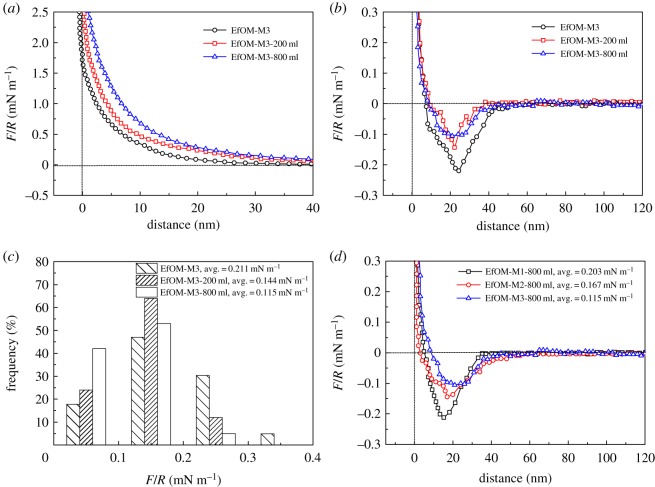
Normalized force (*F*/*R*) versus separation curves between the EfOM-coated probe and the membranes fouled by EfOM: (*a*) approaching curve of M3, (*b*) retracting curve of M3, (*c*) the frequency distribution of the average retraction adhesion force of M3, and (*d*) retracting curve of M1–M3 after filtration of 800 ml of effluent.

The rate of EfOM and membrane fouling was determined by the attachment of EfOM in the bulk solution to the fouling layer. Fouling at this stage was governed by the EfOM–EfOM interactions. Examples of the approaching and retracting force versus separation curves for an EfOM-coated probe and the M3 membrane fouled by EfOM are shown in figure 6. The M3 permeation flux declined rapidly during filtration of 200 ml of secondary wastewater effluent and maintained a steady value during filtration of 800 ml of effluent (figure 2). Based on the filtration data above, the interaction forces between the EfOM-coated probe and the EfOM on the M3 membrane surface during the different filtration processes were measured. Our observations suggested that the membrane was not completely covered with EfOM after filtration of 200 ml of effluent, but was fully covered with EfOM after filtration of 800 ml of effluent. The force versus distance curve for the approach of an EfOM-coated probe to the M3 membrane during the different filtration processes is shown in figure 6*a*. In each case, the force was repulsive at all separation distances. There was no indication of attractive forces at short distances. The range of the measurable interaction increased slightly as the filtration volume increased, as was expected from electrostatic double force. This phenomenon might be interpreted to mean that the surface charge of the fouled membrane could be increased because of EfOM adsorption on the membrane. The zeta potential value of the EfOM solution was –52.3 mV, which could explain the increase in repulsion observed. The adhesion force curves for clean and EfOM-covered membrane regions are presented in figure 6*b*. The adhesion profiles in the clean and covered membrane regions showed distinct differences, with the retraction curves taken on the EfOM-covered areas showing a weaker adhesion force compared with the clean membrane region. The retraction force curves measured on partially covered areas (200 ml) yielded an intermediate adhesion value. During this stage, the membrane fouling behaviour was controlled by both EfOM–membrane and EfOM–EfOM interactions. These results indicated that the interactions between EfOM molecules were much weaker than the interactions between EfOM and the membrane surface. Similar trends for M1 and M2 membrane fouling with BSA solution were observed in this study (data not shown). However, the average adhesion force between EfOM and M1 membrane after filtration of 800 ml of effluent was 0.203 mN m^−1^, while for EfOM–EfOM-covered M2 membrane, it was 0.167 mN m^−1^ (figure 6*d*). These values were a little higher than those obtained for M3 membrane, which might be associated with the hydration of the membrane surface. The QCM-D analysis (figures 3 and 4*a*) showed that the M3 surface had a ‘soft’ open structure layer and the lowest amount of adsorbed EfOM, indicating that more water molecules might be present in the adsorption layer. Thus, the water molecules were less likely to cause an interaction between the EfOM-coated probe and the EfOM adsorption layer. It also indicated that the hydrophilic modification of the membrane could effectively decrease the interactions between foulant and foulant in the later filtration stages.

The data shown in figures 5 and 6 demonstrated that the force versus separation curve could provide additional insights into membrane fouling performance. It was interesting to find that the higher the repulsive force, the lower the adhesion force under these conditions. A high repulsive force could effectively prevent absorption of the foulant onto the membrane. This provided interesting evidence of the importance of the adsorbed water film in those cases where a short-range attraction, as opposed to a completely repulsive interaction, was measured. The adhesion forces between EfOM and membrane were larger than the forces between EfOM and EfOM on the membrane surface, indicating that the membrane fouling largely occurred because of the physico-chemical interaction between EfOM and the membrane rather than the EfOM–EfOM interactions. When combined with the available QCM-D data, there was a close relationship between the magnitude of the adhesion force and the conformation of the EfOM layer. When the adhesion force was low, the structure that formed was non-rigid and open. Thus, further analysis of the force versus distance curve and conformation of the EfOM layer were found to be a useful indicator of the extent to how deposition had occurred on the membrane surface.

### 3.6. Membrane surface morphological analyses

**Figure 7.**
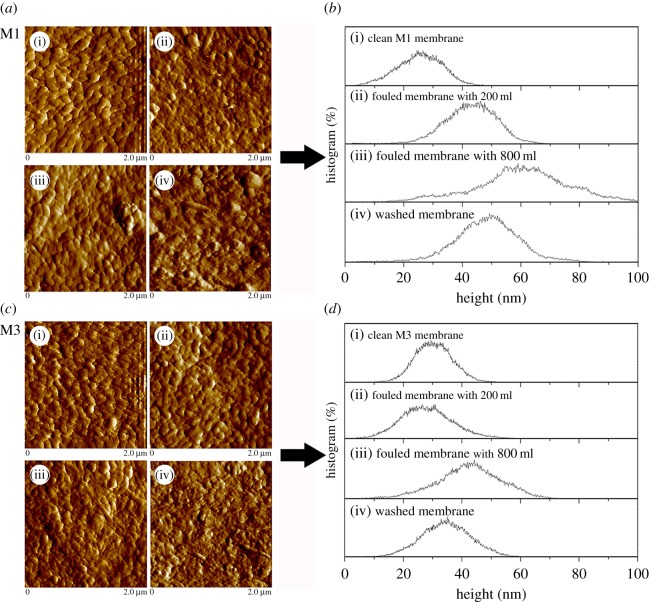
(*a,c*) AFM surface images and (*b,d*) height distribution on the top surface of clean M1 and M3 blended membrane fouled with EfOM: field of view is 2 × 2 µm; (i) clean membrane, (ii) fouled membrane with 200 ml EfOM, (iii) fouled membrane with 800 ml EfOM, and (iv) membrane washed by water rinsing.

Morphological changes during actual membrane filtration processes were investigated using AFM to verify the results of QCM-D and AFM force measurements. As an example of the data obtained, figure 7*a,c* shows the top surface images of M1 and M3 membranes. In the range of the scan area (2 × 2 µm), the *Ra* values of the M1 and M3 membranes were 6.13 and 10.61 nm, respectively. Clean M1 membrane was smoother than clean M3 membrane, indicating less fouling potential. However, the fouled M1 membrane provided a higher topographical view, with the surface of the M3 membrane heavily covered with foulant after filtration of 800 ml of secondary wastewater effluent.

Height distribution on the surface of the membrane, determined using a defined level, can be another useful descriptor for identifying fouling mechanisms [50,51]. Figure 7*b,d* shows the height distributions of M1 and M3 after filtering secondary wastewater effluent. The clean M1 membrane showed a narrow depth distribution with a peak around 25.5 nm. The lightly fouled membrane (200 ml of secondary wastewater effluent) had a broad peak around 43.2 nm that became dominant instead of the peaks around 25.5 nm. When the membrane was heavily fouled (800 ml), a deeper height distribution (around 60.1 nm) was formed, suggesting that accumulation of a cake layer on the membrane surface was significant. When comparing the M1 membrane with the lightly fouled M3 membrane (200 ml of secondary wastewater effluent), the height distribution became broader and an additional peak was seen around 29.1 nm. This may be attributed to an irregular distribution of pores on the membrane surface caused by pore blockage or a widely developed thin surface coverage. The thickness of the cake after filtering 800 ml of secondary wastewater effluent was thinner than that of the M1 membrane. The results indicated that the foulants were not deposited onto the M3 membrane easily and the adsorption rate onto M3 was slower than that of M1. In addition, the –OH-induced membrane surface (M3) could effectively prevent foulant deposition onto the membrane and allow the adsorbed foulants on the membrane to be more readily dislodged by shear force when compared with the M1 membrane. The results were consistent with the QCM-D and AFM force curve observations. These data indicated that with a high affinity interaction between EfOM and membrane, severe fouling occurred. By contrast, when weak EfOM–membrane interactions occurred, a low degree of deformity and a more non-rigid layer structure was formed, which could easily be washed off from the membrane surface.

## 4. Conclusion

This study illustrated the use of QCM-D and AFM for the study of EfOM–SiO_2_-terminated membrane surfaces interactions. The QCM-D and AFM force results indicated that EfOM had a much higher binding affinity towards the hydrophobic compared to the hydrophilic surface, forming a more deformed and rigid structure when adsorbed onto the hydrophobic surface. Elimination of the interaction between EfOM and the membrane could effectively control membrane organic fouling, not only by effectively reducing the amount of EfOM adsorption, but also by changing the structure of the EfOM adsorption layer.

## Supplementary Material

Raw data

## Data accessibility

The datasets supporting this article have been uploaded as part of the electronic supplementary material.
